# Diastrophic dysplasia: prenatal diagnosis and review of the literature

**DOI:** 10.1590/S1516-31802013000100024

**Published:** 2013-04-01

**Authors:** Jonathan Celli Honório, Rafael Frederico Bruns, Luciana Fernandes Gründtner, Salmo Raskin, Lilian Pereira Ferrari, Edward Araujo, Luciano Marcondes Machado Nardozza

**Affiliations:** I Undergraduate Pharmacy Student, Faculdades Integradas do Brasil (UniBrasil), Curitiba, Paraná, Brazil.; II MD, PhD. Adjunct Professor, Department of Gynecology and Obstetrics, Universidade Federal do Paraná (UFPR), Curitiba, Paraná, Brazil.; III MD. Attending Physician, Centro de Diagnósticos (CEDUS), Joinville, Santa Catarina, Brazil.; IV PhD. Associate Professor, Group for Advanced Molecular Investigation (NIMA), Graduate Program in Health Science (PPGCS), Health and Biosciences School (ESB), Pontifícia Universidade Católica do Paraná (PUCPR), Curitiba, Paraná, Brazil.; V PhD. Collaborating Professor, Biomedicine Course, Faculdades Integradas do Brasil (UniBrasil), Curitiba, Paraná, Brazil.; VI MD, PhD. Adjunct Professor, Department of Obstetrics, Universidade Federal de São Paulo (Unifesp), São Paulo, Brazil.; VII MD, PhD, Associate Professor, Department of Obstetrics, Universidade Federal de São Paulo (Unifesp), São Paulo, Brazil.

**Keywords:** Prenatal diagnosis, Osteochondrodysplasias, Ultrasonography, Mutation, Genetic loci, Diagnóstico pré-natal, Osteocondrodisplasias, Ultrassonografia, Mutação, Loci gênicos

## Abstract

**CONTEXT:**

Diastrophic dysplasia is a type of osteochondrodysplasia caused by homozygous mutation in the gene DTDST (diastrophic dysplasia sulfate transporter gene). Abnormalities occurring particularly in the skeletal and cartilaginous system are typical of the disease, which has an incidence of 1 in 100,000 live births.

**CASE REPORT:**

The case of a pregnant woman, without any consanguineous relationship with her husband, whose fetus was diagnosed with skeletal dysplasia based on ultrasound findings and DNA tests, is described. An obstetric ultrasound scan produced in the 16^th^ week of gestation revealed characteristics that guided the clinical diagnosis. Prominent among these characteristics were rhizomelia of the lower and upper limbs (shortening of the proximal portions) and mesomelia (shortening of the intermediate portions). Both upper limbs showed marked curvature, with the first finger of the upper limbs in abduction and clinodactyly of the fifth finger. Molecular analysis using the polymerase chain reaction (PCR) and gene sequencing detected mutations that had already been described in the literature for the gene DTDST, named c.862C > T and c.2147_2148insCT. Therefore, the fetus was a compound heterozygote, carrying two different mutations.

**CONCLUSIONS:**

Prenatal diagnosis of this condition allowed a more realistic interpretation of the prognosis, and of the couple’s reproductive future. This case report shows the contribution of molecular genetics towards the prenatal diagnosis, for which there are few descriptions in the literature.

## RESUMO

**CONTEXTO:**

A displasia diastrófica é uma osteocondrodisplasia causada por mutação homozigótica no gene DTDST (diastrophic dysplasia sulfate transporter gene). As alterações, principalmente nos sistemas esquelético e cartilaginoso, são típicas dessa doença, que tem uma incidência de 1 em 100.000 nascidos vivos.

**RELATO DO CASO:**

Descreve-se o caso de uma mulher gestante, sem relação consanguínea com o seu marido, cujo feto foi diagnosticado com displasia esquelética baseado na ultrassonografia e em testes de DNA. A ultrassonografia obstétrica, realizada na 16^a^ semana, revelou características que guiaram o diagnóstico clínico. Entre elas, se destacam os membros inferiores e superiores com rizomelia (encurtamento das porções proximais) e mesomelia (encurtamento das porções intermediárias), ambos os membros superiores mostraram acentuada flexão, o primeiro dedo em abdução nas extremidades superiores e clinodactilia do quinto dedo. A análise molecular pela reação em cadeia da polimerase (PCR) e o sequenciamento gênico detectou mutações já descritas na literatura para o gene DTDST, chamado c.862C > T e c.2147_2148insCT. Portanto, o feto é um heterozigoto composto, carreando duas mutações diferentes.

**CONCLUSÕES:**

O diagnóstico pré-natal dessa condição permite uma interpretação mais realista do prognóstico e do futuro reprodutivo do casal. Este relato de caso mostra a contribuição da genética molecular no diagnóstico pré-natal, com poucas descrições na literatura.

## INTRODUCTION

Diastrophic dysplasia (DTD) is a type of congenital osteochondrodysplasia with an autosomal recessive inheritance pattern, first described by Lamy and Maroteaux in 1960. In general, osteochondrodysplasias are changes to the skeletal system that produce bone remodeling, cartilage and growth disorders. They are characterized by micromelia dwarfism, progressive scoliosis, bilateral talipes equinovarus, multiple digital deformities (short fingers, synostosis of proximal interphalangeal joints, abnormal thumb insertion and a thumb known as a “hitchhiker”, positioned as in the act of hitchhiking, due to deformity of the first metacarpus), short stature (the reason why the disease is also called diastrophic dwarfism), characteristic ear deformities and, occasionally, a cleft palate. The term diastrophic is appropriate, because it comes from the Greek word diastrophos, which means twisted or bent.^1^^,^^2^

DTD is an unusual type of dwarfism. It has been observed in most white populations, but is notably more prevalent among Finns. In Finland, 1-2% of the population carries a mutation in the DTD gene (DTDST), with an incidence of 1 in 33,000 births. In the United States, the incidence is 1:500,000 births. In 1990, there were 160 Finn patients with a confirmed diagnosis of skeletal dysplasia, while there were fewer than 300 patients in the rest of the world.^3^^,^^4^^,^^5^

During the formation of the skeletal system, most of the bones originate from the cartilage, in a process called endochondral ossification. This process allows elongation and thickening of the bones as the fetus grows, and it continues throughout childhood until bone growth ceases.^6^ In adults, cartilage is present in the ribs, outer ear, airways and joints, in which it has a support function. Cartilage is essential for growth and support of the human skeletal system. Its biological characteristics depend mainly on the cartilage matrix, which is composed of cartilage-specific collagen and sulfated proteoglycans. Proteoglycans are a family of macromolecules formed by chains of core proteins that covalently bind glycosaminoglycans (GAGs), which are large unbranched polysaccharide chains that are classified into four groups: (a) chondroitin sulfate and dermatan sulfate; (b) heparin sulfate and heparin; (c) keratin sulfate; and (d) hyaluronic acid. The first three groups undergo sulfation and bind to proteins to form proteoglycans. The four groups are distributed heterogeneously in various tissues such as cartilage, synovial fluid, connective tissue, skin and lungs.^5^^,^^7^^,^^8^

The diastrophic dystrophy sulfate transporter (DTDST) gene, also known as SLC26A2 (solute carrier family Homo sapiens 26), encodes a membrane sulfate transporter anion protein that is necessary for sulfation of the GAGs that form proteoglycans, which comprise the cartilage matrix. The protein performs exchanges between sulfate and chloride ions. Sulfation of GAGs makes them negatively charged and contributes towards their ability to retain sodium and water, thereby maintaining the hydration of the cartilage matrix and contributing towards its property of impact absorption. Skeletal dysplasia occurs as result of a mutation that changes the process of sulfation, which interferes with endochondral ossification and, hence, the formation of the skeleton and linear growth.^3^^,^^7^^,^^8^^,^^9^^,^^10^

### Molecular characterization of mutations in the DTDST gene

In 1991, Hästbacka et al.^11^ elucidated the structure of the DTDST gene, which consists of three exons. Two of these are coding exons; they are separated by an intron of approximately 1.8 kb and encode a protein of 739 amino acids that forms 12 transmembrane domains and a carboxy-terminal cystoplasmic residue. The gene is located on the long arm of chromosome 5q.31-q.34.^7^^,^^10^ Mutations of the DTDST gene are responsible for four clinical manifestations of chondrodysplasia: “classic” diastrophic dysplasia (DTD), multiple epiphyseal dysplasia 4 (MED 4), atelosteogenesis type II (AO-II) and achondroplasia type 1B (ACG-1B).^2^^,^^3^ ACG-1B is the most severe form of chondrodysplasia and is usually lethal before birth or soon after. It is one of the most severe skeletal disorders in humans, characterized by severe hypodysplasia of the spine, chest and limbs. AO-II is a form of chondrodysplasia with clinical and histopathological manifestations similar to the ones found in DTD, but which are more pronounced. Death often occurs during the neonatal period. MED 4 is considered to be the mildest form and is characterized by joint pain (usually in the hips and knees), deformities of the hands, feet and knees, and scoliosis.^2^^,^^3^^,^^4^

Mutations in the DTDST gene were evaluated by Karnisk through the techniques of direct immunofluorescence, immunoblotting and densitometry, by transfection of mutated genes in human embryonic kidney cells (HEK 293). Correlations of these results with the mutations shown in patients with different clinical forms of chondrodysplasia were evaluated. From this, amino acid substitution mutations in the cytoplasmic domain of the protein, encoded by DTDST gene, were seen to partially maintain protein activity (A715V, R279W, Q454P and C653S) or showed no protein function because of lack of adequate expression in the cell membrane (G678V). Mutations that occurred in the transmembrane domains (DV340, N425D and L483P) or that encoded premature stop codons during translation (R178X, Da1751) were responsible for severe alterations in protein structure, which did not allow transportation of significant quantities of sulfate when expressed in mammalian cells.^12^ Karnisk evaluated the connection between genotype/phenotype and the protein activity encoded by the DTDST gene. Patients with the most severe form (ACG-1B) showed null mutation in both alleles (sulfate transportation rate less than 10%). For moderate-severity clinical forms (AOII and DTD), null mutation was observed in one allele and a mutation that partially preserved the protein function in the other allele. Homozygotes for mutations that retained partial function were characterized by the mild form (MED-4), which expressed protein activity of between 39 and 62%.^12^

It is also important to take into account intron mutations, which were not reported in Karnisk’s study. These can lead to a low rate of messenger RNA (mRNA) expression, through changes to the process of intron excision and, consequently, may reduce the rate of adequate protein formation. Mutation IVS1+2 T > C is an example of a mutation located in intron 1. Patients who are homozygous for this kind of mutation, or who are compound heterozygous with one of the alleles with R279W mutation, show clinical conditions similar to DTD or MED 4.^2^^,^^10^^,^^12^

### Diagnosis

The combination of clinical, radiological and histopathological characteristics in diastrophic dysplasia allows diagnosis at birth. According to a study conducted at the Instituto da Criança, Hospital das Clínicas (HC), Faculdade de Medicina da Universidade de São Paulo (FMUSP), by Gonzales and Marcondes,^1^ the presence of micromelia, congenital clubfoot and digital deformities, especially the thumb in permanent abduction, facilitates recognition of this pathological condition. Birth weight is normal, while stature is greatly diminished (it has been reported that adults with DTD have an average height of 135.7 cm for men and 129.0 cm for women.^1^ In 85% of the cases, the ears may appear suddenly inflamed and swollen over the first weeks of life. This inflammation persists for several weeks and may leave the ear thickened, hard and with irregular morphology, known as a “cauliflower ear”. The external ear canal is narrow, but generally the hearing is not affected. The head and skull are normal, but the face tends to be square, with narrow nose at the proximal end and large at the middle portion. A cleft palate is found in approximately one quarter of the cases.^1^^,^^5^

Prenatal diagnosis is possible by means of ultrasound and molecular analysis. Ultrasound in the second trimester allows suspicion of the disease when the limbs are shorter than normal. One important characteristic observed in fetuses with diastrophic dysplasia is the preserved relationship between the thoracic and waist circumferences. Moreover, the presence of the first finger in abduction is quite specific.^13^^,^^14^ DNA analysis can be done by extracting genetic material from fetal cells obtained by chorionic villus sampling or amniocentesis. Molecular analysis on the parents is done through collection of genetic material from leukocytes.

Hästbacka et al.^15^ used DNA markers to predict the genotypes of five fetuses in families with previous histories of DTD. The results predicted that three would not be affected and two would. These results were concordant with those obtained from ultrasound, and the fetal phenotype was correctly predicted in all cases. DNA analysis is the most reliable means for prenatal diagnosis in the first trimester of pregnancy.

### Prognosis and treatment

During the neonatal period, the mortality rate is high (25%), usually caused by airway obstruction. If patients survive this period, the prognosis is favorable. Their mental and sexual development are normal.^2^^,^^7^

The treatment for patients with diastrophic dysplasia consists of physiotherapy and other correlated therapies that help to improve mobility. Surgical treatment of clubfoot is indicated when the deformity makes walking impossible. The rate of spontaneous correction of cervical kyphosis is high, and cervical spinal surgery among children may be restricted to individuals with clinical or neurophysiological evidence of spinal shock. According to Helenius et al.,^16^ arthroplasty is indicated for “relatively young adults” with premature degenerative arthrosis. Clinicians should monitor patients’ spinal curvature annually, and their habits in order to avoid development of excess weight (obesity), so that the patients’ joints are not subjected to unnecessary exertion.^2^

## OBJECTIVES

This study aimed to evaluate the ultrasound and molecular aspects of the prenatal diagnosis of diastrophic dysplasia, with a review of the literature on this genetic disorder.

### Sources

For the review, we used University textbooks relating to this topic and researched in the PubMed/Medline, Embase and Lilacs databases. The search in PubMed/Medline and Embase were based on key words such as diastrophic dysplasia, prenatal, pregnancy, abduction of the first finger, ultrasound and DTDST gene. The search in Lilacs was based on key words such as displasia fibrosa óssea, displasia fibrosa ósea, diastrophic dysplasia, (diagnóstico pré-natal, prenatal diagnosis, atención prenatale) and (frequencia do gene, frecuencia de los genes, mutation, mutação).

The patient of this case report agreed to participate in this study, through signing a free and informed consent statement. This project was approved by the Research Ethics Committee of Faculdades Integradas do Brasil (UniBrasil), Curitiba, Paraná, in accordance with the regulations of the National Research Ethics Commission (Comissão Nacional de Ética em Pesquisa, Conep), through opinion report 035/2009.

## CASE REPORT

**Figure 1.**
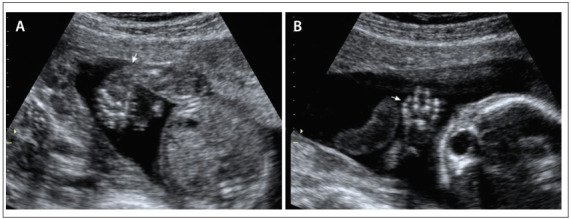
Prenatal ultrasonography findings of diastrophic dysplasia: (a) clubfoot; (b) fifth finger with clinodactyly.

The patient AJDR was 29 years of age (G3, P0, A2), and was seen at 16 weeks and six days of gestation. She and her husband were phenotypically normal and not consanguineous. The patient said that she had not used warfarin during the first trimester of pregnancy. The routine ultrasound revealed that the fetus had short limbs, and the patient was then referred for an evaluation with a geneticist and specialist in fetal medicine. Detailed ultrasound examination (described below) showed signs suggestive of DTD, and amniocentesis was then performed to search for possible presence of mutations in the DTDST gene. Fetal morphological analysis revealed that the ossification of the skull and brain structure appeared to be normal. Although the lips and nose were normal, the facial profile showed micrognathia and the nasal bone was at the 10^th^ percentile for the gestational age. The heart examination was unremarkable. In both the upper and the lower limbs, rhizomelia (shortening of proximal portions) and mesomelia (shortening of medial portions) were observed. Both upper limbs showed marked curvature (campomelia). In the upper limbs, the thumb was observed to be in abduction, with clinodactyly of the fifth finger (Figure 1, above). No other abnormalities were detected. The findings were compatible with fetal skeletal dysplasia.

**Figure 2.**
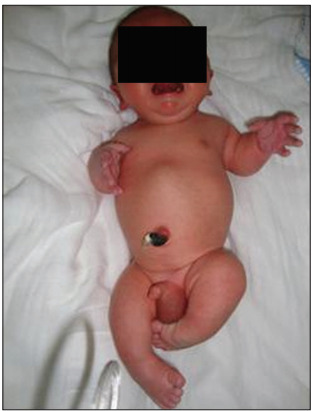
Newborn with diastrophic dysplasia: the first finger can be seen to be in abduction, which is a typical alteration of this pathological condition.

The child of this case (a boy) was born on November 28, 2009, weighing 2.895 kg and with a length of 41 cm (Figure 2). He presented some difficulty in breathing that resolved without the need for mechanical ventilation. At first, the feet were not so crooked (equinovarus feet) and the initial treatment was with correction boots. As expected, the thumb was in abduction and the limbs were shorter. He was also born with hemangioma on the nose and forehead, another common finding in this dysplasia. No dislocations were observed in the clinical examination. Thus, it was believed that, as expected, the prognosis would be good.

**Figure 3.**
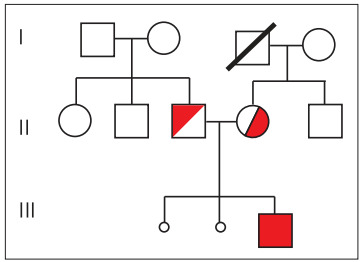
Pedigree of the family of this study.

After the suspicion of a probable case of diastrophic dysplasia had been raised from the ultrasound image, the pregnant woman was referred to a genetic counseling center and molecular genetics laboratory for a prenatal molecular diagnosis to be made and for genetic counseling to be offered. According to the family pedigree (Figure 3), the parents were carriers of the DTDST gene (line II in Figure 3). The fetus was a compound heterozygote, carrying two different mutations (line III). The black balls (line III) indicate two previous miscarriages. The patient said that she had one brother and that her father had died. The husband had a brother and a sister, and both parents were alive. There was no reported family history of skeletal dysplasia in either of the families.

The patient had had two previous miscarriages. When she was at 20 weeks of gestation, amniocentesis was performed in order to carry out genetic tests. Through this procedure, amniocyte DNA was extracted and DTDST locus amplification was performed by means of PCR on three coding exons from the DTDST gene. Two mutations were detected: the transition c.862C > T (R279W) in exon 3, converting the arginine codon (CGG) to tryptophan codon (TGG); and c.2147_2148insCT in exon 3, in the DTDST gene. The second of these mutations, which involves insertion of two bases, causes a change in the reading frame and may result in aberrations in mRNA splicing. Both of these mutations had previously been described in the literature as pathogenic mutations of the DTDST gene.^10^

## DISCUSSION

In this case report, the genetic pathological condition was identified in one allele of the DTDST gene in each member of this phenotypically normal couple: the father was heterozygous for the mutation c.862C > T and the mother was heterozygous for the mutation c.2147_2148insCT.

Differentiation of DTD from superficially similar forms of dwarfism, especially achondroplasia, is important because of the greater morbidity, greater difficulty in case management and poorer reproductive prognosis for the parents in cases of DTD. Achondroplasia is a form of dwarfism produced by changes in growth due to deficiency of endochondral ossification, caused by mutation in the gene located at 4p16.3, which is responsible for synthesis of receptor 3 for fibroblast growth factor. A patient with achondroplasia, born from normal parents, is usually the result from a new mutation. The risk of recurrence is very low in this, while in diastrophic dwarfism it is 25%. Several errors in genetic counseling have been committed because of confusion between these two diseases. Recently, with the advent of prenatal molecular diagnosis, there has been a reduction in diagnostic errors. It has been recommended that families should be assisted through genetic counseling and that appropriate clinical measures should be established early on.^2^

According to Rossi and Superti-Furga, the mutation c.862 C > T (R279W) is the most common mutation in Caucasians. This is characterized by substitution of a cytosine by a thymine, thus causing a reading change from the amino acid codon arginine to a tryptophan. Homozygotes of this mutation tend to have the milder manifestations of dysplasia (MED4), while retaining some residual activity. Since the mutation c.2147-2148insCT, common in French descendants, encodes a premature stop codon during translation, this mutation in homozygous cases is related to achondroplasia type 1B.^10^

The fetus in the present case was diagnosed with a “classical” form of diastrophic dysplasia, showing a mutation that retained partial activity (R279W) and a mutation that led to loss of function (c.2147-2148insCT, a null mutation). This genotype suggested that 50% of the protein production had retained its activity, which agrees with data in the literature confirming that individuals with DTD or AO-II (classical) have a mutation in one allele that retains partial activity, with loss of protein function by the other allele.^10^^,^^12^

As shown in Table 1, there have only been seven case reports of DTD diagnosed by means of prenatal ultrasound.^1^^,^^3^^,^^15^^,^^17^^,^^18^^,^^19^^,^^20^ Moreover, the present case report is only the second in the literature in which prenatal ultrasound and molecular DNA analysis were used for diagnosing DTD.^15^ Unlike the study by Hästbacka et al.,^15^ in which five fetuses from families with previous histories of DTD were assessed, in our case report the couple did not have a consanguineous relationship and there was no previous history of DTD in their families.

**Table 1.** Systematic review of the literature*

Database	Search strategy	Results
Medline/PubMed	(Diastrophic dysplasia) AND (Prenatal diagnosis) AND (Abduction of the first finger) AND (Ultrasound) AND (DTDST gene)	4 reviews 4 original articles 5 case reports
Embase	(Diastrophic dysplasia) AND (Prenatal diagnosis) AND (Abduction of the first finger) AND (Ultrasound) AND (DTDST gene)	No original articles, case reports or review articles
Lilacs	((Displasia fibrosa óssea) OR (Diastrophic dysplasia)) AND ((Diagnóstico pré-natal) OR (Prenatal diagnosis) OR (Atención prenatale)) AND ((Frequencia do gene) OR (Frecuencia de los genes) OR (Mutation OR Mutação))	2 case reports 1 review

^*^The search in these databases was conducted on April 2, 2011.

## CONCLUSION

Prenatal diagnosis of diastrophic dysplasia during the second trimester of pregnancy has fundamental implications for the prognosis, and enables earlier clinical guidance and genetic counseling. Identification of the DTDST gene in the 1990s has make molecular diagnosis reliable for most families with a positive history, and even for families without other family cases, as shown in this report. We emphasize that this study is important because it presents a case in which the diagnosis was confirmed prenatally through molecular analysis, given that in the literature there is only one other case report of a diagnosis confirmed by prenatal molecular analysis.^15^
